# Patient Attitudes toward Gestational Weight Gain and Exercise during Pregnancy

**DOI:** 10.1155/2019/4176303

**Published:** 2019-09-17

**Authors:** M. L. Lott, M. L. Power, E. G. Reed, J. Schulkin, A. D. Mackeen

**Affiliations:** ^1^Maternal-Fetal Medicine, Advocate Lutheran General Hospital, St. Charles, IL, USA; ^2^Division of Maternal-Fetal Medicine, Women's and Children's Institute, Geisinger, Danville, PA, USA; ^3^American College of Obstetricians and Gynecologists, Washington, DC, USA; ^4^Smithsonian National Zoological Park and Conservation Biology Institute, Washington, DC, USA; ^5^University of Washington School of Medicine, Seattle, WA, USA

## Abstract

Body mass index (BMI) and gestational weight gain (GWG) are important factors for neonatal and maternal health. Exercise helps women moderate their BMI and GWG, and provides health benefits to mother and child. This survey study assessed patients' perceptions of counseling they received during pregnancy, their sources of information about GWG, and their attitudes toward exercise during pregnancy. We distributed an anonymous survey to 200 pregnant women over the age of 18 at a tertiary care center in Danville, Pennsylvania. Survey questions included demographics, discussions with medical providers regarding GWG and exercise, and their exercise habits before and during pregnancy. 182 women (91%) responded. Most reported their provider discussed weight and diet (78.8%), expected GWG (81.6%), and exercise during pregnancy (79.8%); however, 28% of obese women and 25% of women who did not plan to exercise during pregnancy reported not receiving exercise counseling. Approximately 20% of women did not plan to exercise during pregnancy. Women decreased the number of days per week they exercised (40.6% with 3 or more days prepregnancy versus 30.7% during pregnancy, *P* = 0.002). Some patients who did not exercise prior to pregnancy (12%) expressed interest in a personal training session. Among women in the eight month or later, 42.4% were above GWG recommendations. Our study found barriers to adequate activity during pregnancy; 20% of pregnant women not receiving/remembering counseling regarding exercise. Interest in personal training from patients that did not exercise suggests they would benefit from increased efforts to encourage physical activity. Exercise and GWG counseling based in medical science as well as patient psychological needs will help efforts to reduce GWG and improve pregnancy outcomes.

## 1. Introduction

Maternal body mass index (BMI) and gestational weight gain (GWG) are important factors for neonatal and maternal health. Underweight women who do not gain enough weight during pregnancy are at risk of small for gestational age neonates and preterm birth [1–4], birth outcomes associated with poor health later in life for the neonate. High maternal BMI and excessive GWG are independent risk factors for increased neonatal adiposity [5]. Excessive GWG is also a risk factor in all BMI categories for fetal macrosomia, cesarean delivery, postpartum weight retention [6], and future obesity of both mother and child [7–10]. In 2009, the Institute of Medicine (IOM) published revised guidance on GWG that accounted for the mother's prepregnancy BMI with regard to recommendations for total GWG and weekly weight gain during the second and third trimesters [6]. In 2013 the American College of Obstetricians and Gynecologists (ACOG) endorsed the 2009 IOM GWG recommendations [11].

Exercise can be an important component of lifestyle behaviors that help women moderate their BMI and GWG, and provides additional health benefits to mother and child. Exercise during pregnancy improves or maintains cardiovascular fitness, reduces the risk of gestational diabetes, hypertensive disorders, macrosomia, and cesarean deliveries [12, 13], and enhances psychological well-being [14]. Exercise during pregnancy does not increase the risk of preterm birth or shorten gestation [12]. Indeed, in overweight and obese women, exercise during pregnancy is associated with a decreased risk of preterm birth [15]. Neonates of women who reduced their exercise levels at 20 weeks of gestation were more likely to have an adiposity above the 90^th^ percentile [16]. Exercise in pregnancy has also been shown to decrease total labor time [17].

ACOG recommends that all women with uncomplicated pregnancies engage in moderate intensity physical activity 30 min per day most days of the week during pregnancy, in the absence of maternal contraindications [18]. However, studies have found that only a minority of pregnant women achieve the recommended level of activity, with a consistent decrease over pregnancy [19, 20]. Being inactive has become a leading risk factor for mortality and morbidity inside and outside of pregnancy [20].

ACOG and the American Academy of Pediatricians recommend obstetric providers offer education and counseling regarding GWG, diet, and exercise as an integral aspect of routine prenatal care [21]; however, the effectiveness of current provider counseling practices is uncertain. A qualitative study of providers found that providers did not place a priority on appropriate weight gain, had few resources for patients, and believed that any advice they gave was unlikely to be followed [22]. One year after ACOG endorsed the IOM recommendations for GWG, a survey of practicing ACOG Fellows found that almost one-in-five were unaware of the recommendations and roughly half were not confident in their ability to influence GWG [23]. In a later study, although most providers believed that excessive GWG was a major health concern in their practice, 40% were not confident in their ability to affect their patients' GWG [24]. From the patient perspective, two recent studies found that about one-third of the enrolled pregnant women did not recall receiving advice regarding GWG [25, 26]. Overweight and obese women were either more likely not to recall receiving guidance or more likely to have received advice not consistent with the IOM guidelines [25, 26].

 The purpose of this survey study is to assess the perception of patients regarding the counseling they received during pregnancy for GWG and exercise, their sources of information about GWG, and to identify patient attitudes toward exercise during pregnancy as compared to their prepregnancy ideals and activity level. Specifically, we are interested in the willingness of patients to consider further education regarding exercise during pregnancy including personal training sessions. We examine the self-reported activity exercise level from before and during pregnancy and reasons for any change, such as unwillingness to participate in activities, fear of exercise during pregnancy, uncertainty regarding the safety of exercise during pregnancy or direct instruction by an OB provider that they are restricted from exercise during pregnancy.

## 2. Materials and Methods

An anonymous survey was distributed to 200 pregnant women over the age of 18 receiving prenatal care at a tertiary care center in Danville, Pennsylvania. Survey questions included demographic information (age, height, prepregnancy weight, current weight, education level, ethnic background, and insurance information), prepregnancy perception of obesity and diabetes, perceptions of discussions with medical providers regarding GWG and the consequences of inappropriate GWG, and exercise habits both before and during pregnancy. For survey responses regarding types of exercise, the following definitions were used: Low-impact cardiovascular activity included walking, elliptical, cycling, or swimming. High-impact cardiovascular activity included running, cross-training, or step aerobics. Weight training included free weights and weight machines. Surveys also inquired into which sources of pregnancy information the patient considered most helpful in understanding GWG. The patients were instructed to complete the survey questions and then seal their responses in the provided envelope. The envelopes were collected and sent to the Research Department at the American College of Obstetricians and Gynecologists for review and analysis. The study was approved by the Geisinger Institutional Review Board and classified as exempt.

Data were analyzed using a personal computer-based statistical package (IBM SPSS 24.0; IBM Corp, Armonk, NY). Descriptive statistics were computed for the measures. Missing values were excluded when calculating frequencies. Patients' prepregnancy BMI was calculated from their self-reported height and prepregnancy weight. Weight gain at the time of the survey was calculated by subtracting their prepregnancy weight from their self-reported current weight. Continuous parameters are presented as mean ± SEM. Chi-square was used to test associations between categorical variables. Significance was set at *P* < 0.05.

## 3. Results

Demographic information for the 182 women (91%) that completed and returned the survey are given in Table 1. The patient population studied had an average age of 29.2 years (SEM = 0.4 years), with a mean prepregnancy BMI of 29.6 ± 0.6 kg/m^2^. A majority were either overweight or obese (Table 1). Almost all (93.9%) the respondents were white. Almost all were either married (62.1%) or living with a partner (29.1%). Most had private health insurance (60.8%). The patients were, on average, in their seventh month of pregnancy (range of second month to tenth month). Almost forty percent (39.7%) reported their provider was a medical doctor, with 23.5% reporting a nurse midwife, and 8.4% reporting a nurse practitioner as a provider (11.7% responded they were not sure who their provider was). Thirty of the women reported having two or more providers (16.8%).

Most women reported that their health care provider had discussed their weight and diet (78.8%), the expected amount of weight they should gain (81.6%), exercise and physical activity during pregnancy (79.8%), and the importance of controlling their blood sugar during pregnancy (70.4%). Fewer, but still a majority of women reported being told about possible harms to their baby (67.0%), possible harms to themselves (65.4%), and possible problems with delivery (64.6%) from excessive GWG. A discussion of possible harms to their baby from too little GWG was reported by 58.7%. A majority of women considered their obstetrician (76.4%) or another health care provider (62.6%) a good source of information regarding GWG. Neither the patient's prepregnancy BMI nor the month of pregnancy was associated with whether she recalled being counseled by her provider on these topics, and there was no difference in the patients' recall of advice/counseling based on type of provider. Insurance status made no difference in recall of counseling for all questions except the amount of weight the patient should expect to gain during pregnancy. Respondents with private insurance were more likely to recall counseling on GWG expectations than respondents with Medicaid or no insurance (86.4% versus 69.8%, *P* = 0.017).

Sixty-six patients were in their eighth month or later of pregnancy. This group's demographics were no different than the overall study population's demographics (primary provider, race, insurance, education, or relationship status). Within this subset, 42.4% of women were above recommendations for GWG, based on their self-reported height and prepregnancy and current weights. Overweight women were the most likely to have excessive GWG (72.7% versus 26.1% and 43.8% for normal weight and obese women, respectively, *P* = 0.040). There was no difference in the recall of counseling by providers by either BMI or GWG category. Similarly, there was no difference in BMI or GWG category based on primary provider, race, relationship, or education (data not shown). Compared to patients with Medicaid or no insurance, patients with private insurance were less likely to have GWG below recommendations (12.5% versus 46.7%) and more likely to have GWG within or above recommendations (40.0% versus 13.3%, 47.5% versus 40.0%, *P* = 0.015).

Prepregnancy, only about one in five of the women (19.8%) reported not regularly engaging in exercise. About two-thirds (66.5%) reported engaging in low-impact cardio exercise prepregnancy, with fewer engaging in high-impact cardio (17.0%) or weight training (17.6%). The most common response was engaging in low-impact cardio only (62.4%); very few reported engaging in all three exercise types (4.6%). Prepregnancy, obese women were more likely than overweight or normal weight women to engage in low-impact cardio (79.7% versus 60.4%, *P* = 0.008) and less likely to engage in high-impact cardio (4.1% versus 30.8%, *P* < 0.001) or strength training (9.5% versus 27.5%, *P* = 0.003) (Table 2). Also prepregnancy, patients with Medicaid or no insurance were less likely to engage in high-impact cardio than patients with private insurance (0.0% versus 24.8%, *P* < 0.001), and less likely to engage in weight training (7.5% versus 23.8%, *P* = 0.019).

During pregnancy, the proportion of women engaging in low-impact cardio increased (79.1%), with 70.4% of the women reporting low-impact cardio as their only exercise. The proportion engaging in high-impact cardio (4.4%) and weight training (9.9%) decreased substantially (Table 2). In general, the women decreased the number of days per week they exercised (40.6% reported exercising 3 or more days prepregnancy versus 30.8% during pregnancy, Table 2, *P* = 0.002). During pregnancy, there was no difference in the proportion of women engaging in low-impact cardio by BMI category, however, obese women were still significantly less likely to engage in high-impact cardio or strength training (Table 2).

Among the aforementioned 66 women in their eighth month or later in pregnancy, 20.9% reported not exercising prepregnancy and 29.9% did not plan to exercise during pregnancy. Before pregnancy 38.8% of these 66 patients exercised 3 or more days per week, but during pregnancy this figure reduced to 23.9%. Exercise status before pregnancy and plans to exercise during pregnancy made no difference to whether their GWG was within or above recommendations.

Of the patients that did not plan to exercise during pregnancy, reasons reported for not exercising included: being unsure if they were allowed to exercise (8%), being afraid to exercise during pregnancy (8%), not liking to exercise (29%), being specifically instructed to not exercise by their provider (2%), and 56% gave other reasons for not exercising such as illness, injuries, lack of time in the day, heat, or cold. Nineteen percent of patients reported they were not counseled regarding exercise in pregnancy, with 32.4% of patients that did not exercise prior to pregnancy reporting they did not receive counseling. Twenty six percent of obese women and 21.2% of women who did not plan to exercise during pregnancy reported they did not receive counseling. About 32% of patients who exercised and 12.9% of those who did not exercise prepregnancy expressed interest in meeting with a personal trainer for an exercise counseling session. Of those interested in personal training, 29.4% reported not receiving any exercise counseling from a provider.

## 4. Discussion

Pregnancy is a time in which women that may not otherwise seek medical care have regular contact with health care providers, providing an opportunity for health interventions that may not have otherwise occurred. This includes opportunities for counseling regarding healthy activity and lifestyle changes to optimize maternal health and pregnancy outcomes. Pregnancy can be considered as a “teachable moment” that may further mold a woman's dietary and lifestyle choices beyond the puerperium into lifelong improvements in overall health and fitness [27].

Obesity remains a growing public health concern, particularly among pregnant women. A majority of our study population were overweight or obese by self-report. Inappropriate GWG also continues to be a serious public health concern [28], and a large proportion of the women in this study had excessive GWG despite a majority self-reporting receiving counseling and information from their health care provider. This result is consistent with a 10-year retrospective study of GWG in this population using medical records that found that a majority of women gained above recommendations, with no change in the pattern over the 10 years [29]. Previous published findings indicate the tools available to providers are not sufficient to effect positive change in GWG outcome [26, 30]. Obstetric providers need increased training to effectively and efficiently deliver counseling regarding the health benefits of appropriate GWG, healthy activity, and duration and intensity of exercise. Once consistency of provider counseling is demonstrated, we can then focus on the tools available to patients to allow them successful achievement of GWG and fitness goals. Delgado et al. reported differences in physician versus patient perceptions of counseling, as well as utility of various methods of patient education including posters, hand-outs, and phone applications [31]. Seventy seven percent of patients found information regarding local fitness resources useful as compared to half of providers perceiving them as helpful[31]. Provider counseling must be grounded in both medical science and patient preferences and behavior.

Overweight and obesity-specific counseling and recommendations may be a helpful manner by which at-risk populations can begin to improve intrapartum, pregnancy and neonatal outcomes, as well as long term maternal health. Patients in all BMI categories were equally as likely to have received GWG and exercise counseling, but still overweight patients were most likely to exceed GWG recommendations. Obstetric providers can prescribe exercise in overweight and obese patients through an effective approach tailored toward previously sedentary women to slowly and safely reintroduce physical activity into their everyday lives [32]. Our survey showed one in five respondents were not exercising before pregnancy and that 32% of this group did not receive exercise counseling. This may represent a lost opportunity to encourage life-long healthy behaviors as well as potentially moderate GWG.

Our results are consistent with findings from previous studies assessing patient perception of exercise and barriers to physical activity in pregnancy. Petrov Fieril et al. conducted face-to-face interviews of 17 pregnant patients in order to describe experiences of exercise while pregnant [33]. Fourteen of the 17 exercised regularly before pregnancy, performing highly repetitive resistance training in a group setting on a regular basis. The positive impacts of exercise were commonly discussed, as were some limitations to active participation such as concern regarding the effect on the fetus, physical limitations, and an overall lack of knowledge regarding physical activity in the gravid state [33]. Similarly, our patients also reported a lack of knowledge of acceptable exercise during pregnancy, as well as psychological concerns, including fear and dislike of exercise.

A majority of respondents planned to exercise during pregnancy, most of whom planned to engage in low-impact cardio activity, which has been shown to reduce GWG and gestational diabetes [13, 34]. However, 20% of patients did not plan to exercise; 29% of those reported it was because of a dislike of exercise, pointing to a need for further study and incorporation of emotional drivers of exercise intent into interventions. Gustafsson et al. evaluated the impact of exercise on the quality of life of patients that were randomized to participate in thrice weekly moderate intensity exercise [35]. The number of women not meeting the ACOG standards for exercise in pregnancy is consistent with our findings. Interestingly, the overall Psychological General Well-being Index (PGWBI) scores were not different between groups and exercise did not appear to positively impact psychological wellbeing or self-perceived health [35]. Rauff and Downs found in their prospective study that the primary predictor for exercise in pregnant women was intention, and that overweight and obese participants had lower attitudes and intention toward exercise compared to normal weight participants [36]. The study's similar population demographics to ours and location in Central Pennsylvania are likely indicative that exercise intention is an influence in our population's responses.

In a systematic review and meta-analysis, Choi et al. found supervised physical activity showed less GWG than controls, while unsupervised physical activity demonstrated a nonsignificant increase in GWG as compared to controls [37]. The studies that included unsupervised physical activity did offer counseling regarding recommended physical activity during pregnancy but did not prescribe patient-specific regimens, which indicates that a more intensive and individualized approach to exercise in pregnancy, rather than generalized recommendations, may be a crucial aspect of reducing GWG [37]. It is concerning that among our respondents in their eighth month of pregnancy or later, exercise status before and during pregnancy had no influence on GWG; however, many factors not included in our survey, such as diet, could have influenced GWG outcomes. Although exercise did not help moderate GWG for these women, they could have experienced many other benefits of exercise during pregnancy such as reduced risk of preterm birth [15] and decreased labor time [17].

Our results reflect a willingness to counsel with a physical trainer during pregnancy, as well as a reluctance to start an exercise intervention due to fear or lack of understanding of appropriate activity during pregnancy. The direct supervision and feedback provided by a physical trainer can offer a patient reassurance and motivation as well as an individualized fitness routine that can be tailored to the changing gravid abdomen and exercise tolerance to improve pregnancy outcomes and decrease GWG. A referral to a physical trainer should be considered for all women in pregnancy in whom exercise is not contraindicated.

Pregnancy is an optimal time for behavioral interventions guided by policy, because of the regular contact with health care providers women receive during pregnancy. However, IOM recommendations themselves are not enough to affect GWG practices [38], and internationally many countries have no policies in place for excessive GWG [39]. Our study implicates that current GWG and exercise counseling practices are not sufficient, as a majority of patients reported receiving counseling on both, yet were still above IOM GWG recommendations. Health care providers often overestimate the percentage of their patients with GWG within recommendations, and sometimes underestimate the percentage of their patients with GWG above recommendations [24]. Additionally, health care providers understand the benefits of exercise during pregnancy but are less well versed in the appropriate ACOG recommendations for exercise [40]. Provider counseling methods should be revised to more prominently include patient preferences, as personal intent is critical to achieving exercise participation results, and therefore aid in appropriate GWG [36].

Counseling must also be behavioral change focused, as behavioral strategies play an important role in successful GWG interventions [41]. Women who are more knowledgeable about their BMI category and GWG recommendations prepregnancy are more likely to gain within GWG recommendations [42, 43]. Similarly, women must be knowledgeable about appropriate exercise options during pregnancy in order to meet ACOG activity recommendations [18]. A small but noteworthy group of our respondents reported confusion over what exercise practices were safe to pursue during pregnancy, which is consistent with previous studies of demographically similar populations [44]. Practitioners should use counseling practices that outline personal GWG goals and safe options for exercise in a format, such as pamphlets/brochures, preferred by patients [31]. Exercise regimes individualized for each patient would be the optimal practice, as unsupervised and general exercise plans are less effective in helping achieve appropriate GWG [37]. Equally important but beyond the scope of our study, policies and counseling practices should address women's health preconception, specifically achieving a normal BMI, as ill-health preconception leads to increased complications when pregnancy does occur [45].

This study has several limitations. Due to the anonymous nature of the survey, both height and weight were patient reported information, thus making confirmation of BMI and weight gain uncertain. Exercise patterns were all self-reported and we cannot distinguish between actual exercise patterns as they occurred from reported plans to exercise. For example, we assumed that the 66 patients who were in their eighth month or later of pregnancy were reporting their actual exercise behavior, as it would be unreasonable to assume that if they had not been exercising prior that they would begin at the end of pregnancy. Another limitation is that our patient population is predominantly Caucasian in a rural area, which leads to the inability to generalize our results to other populations. We did not include a referral to a physical trainer to our patients as part of our study, although the anonymity of the survey study would not have allowed evaluation of patient compliance or the effect of training. The rural location of the study hospital and limited access to health clubs and personal trainers or other fitness experts are an inherent limitation to our population, which may also have impacted patient reported exercise habits during pregnancy. Limited access to fitness facilities and personnel due to location, as well as cost inhibitors to fitness are concerns for health during pregnancy. In our study, women with private insurance were more active and had better GWG patterns than women with Medicaid or no insurance, showing that financial challenges could be a factor of exercise and GWG.

Strengths of our study include anonymous results, allowing patients to be honest in answering questions regarding activity levels without fear of judgment. Many studies have discussed emotional ties to exercise, particularly during pregnancy, so patient anonymity and the security to be honest are critical [35, 36]. Additionally, introducing the option of further counseling with a physical trainer allowed for us to ascertain patient receptiveness to exercise intervention beyond their current tendencies, as well as identify patient groups that may be most likely to benefit from this kind of service.

## 5. Conclusions

It is encouraging that over 80% of women in this population, in which almost 2/3 were overweight or obese, planned to exercise during pregnancy. Most of the planned activity was low-impact cardiovascular activity, which has been found to reduce excessive GWG. However, our study demonstrates many barriers to adequate activity during pregnancy. It is troubling that 20% of pregnant women were not adequately counseled regarding exercise, including 28% of obese women and 25% of women who did not plan to exercise during pregnancy. Twelve percent of patients who did not exercise prior to pregnancy expressed interest in a personal training session, suggesting that some women who may not otherwise exercise regularly are interested in and would benefit from further efforts, encouragement, and education regarding physical activity. Exercise and GWG counseling based in medical science as well as patient psychological needs will help efforts to reduce GWG and improve pregnancy outcomes.

## Figures and Tables

**Table 1:** Demographic information for the 182 women that completed the survey.

Demographic	*N*(%)
*Education*	*n* = 182
Less than high school diploma	2 (1.1)
High school diploma	46 (25.1)
Some college	40 (21.9)
College graduate	67 (36.6)
Graduate/Professional degree	27 (14.8)

*Race*	*n* = 181
White	170 (93.9)
Black or African American	3 (1.7)
Asian/Hawaiian/Pacific Islander	2 (1.1)
Latina	0 (0)
American Indian or Alaska Native	0 (0)
Other	0 (0)
Two or more selected	6 (3.3)

*Relationship status*	*n* = 182
Married	113 (62.1)
Not married but living together	53 (29.1)
In relationship, not living together	8 (4.4)
Not in a relationship	8 (4.4)

*Insurance*	*n* = 181
None	9 (5.0)
Private health insurance	110 (60.8)
Medicaid/Medicare	35 (19.3)
Military health care	4 (2.2)
Other source	19 (10.5)
Two or more selected	4 (2.2)

*Patient's obstetric care provider*	*n* = 179
Medical Doctor	71 (39.7)
Nurse Practitioner	15 (8.4)
Certified Nurse Midwife	42 (23.5)
Not sure	21 (11.7)
Multiple providers	30 (16.8)

*Patient's BMI category*	*n* = 182
Underweight	2 (1.1)
Normal weight	62 (34.1)
Overweight	34 (18.7)
Obese	78 (42.9)
Unknown	6 (3.3)

**Table 2:** Counseling and patient exercise habits prepregnancy and during pregnancy by BMI.

	All	Normal BMI	Overweight BMI	Obese BMI
	*N*(%)	*N*(%)	*N*(%)	*N*(%)
Received counseling regarding exercise	142 (78.0%)	50 (83.3%)	28 (84.8%)	57 (74.0%)

Desired to meet with personal trainer	49 (26.9%)	11 (18.0%)	11 (35.5%)	26 (34.2%)

*Prepregnancy*				
Exercised before pregnancy	144 (79.9%)	49 (80.3%)	27 (79.4%)	60 (77.9%)
Exercised 3+ days per week before pregnancy	74 (40.6%)	30 (49.2%)	14 (41.2%)	25 (32.5%)
Engaged in low-impact cardiovascular	121 (66.5%)	35 (59.3%)	20 (62.5%)	59 (79.7%)
Engaged in high-impact cardiovascular	31 (17.0%)	18 (30.5%)	10 (31.3%)	3 (4.1%)
Engaged in weight training	32 (17.6%)	16 (27.1%)	9 (28.1%)	7 (9.5%)

*Pregnancy*				
Plan to exercise during pregnancy	144 (79.1%)	48 (77.4%)	24 (72.7%)	62 (80.5%)
Plan to exercise 3+ days per week	56 (30.8%)	23 (37.1%)	8 (24.2%)	21 (27.3%)
Will engage in low-impact cardiovascular	144 (79.1%)	50 (80.6%)	25 (75.8%)	62 (81.6%)
Will engage in high-impact cardiovascular	8 (4.4%)	6 (9.7%)	2 (6.1%)	0 (0.0%)
Will engage in weight training	18 (9.9%)	10 (16.1%)	5 (15.2%)	3 (3.9%)

## Data Availability

The survey data used to support the findings of this study are available from the corresponding author upon request.
